# A Platform for Innovation and Standards Evaluation: a Case Study from the OpenMRS Open-Source Radiology Information System

**DOI:** 10.1007/s10278-018-0088-5

**Published:** 2018-05-10

**Authors:** Judy W. Gichoya, Marc Kohli, Larry Ivange, Teri S. Schmidt, Saptarshi Purkayastha

**Affiliations:** 1 Department of Radiology, Indiana University School of Medicine, 550 N. University Blvd. Room 0641, Indianapolis, IN 46202-2879 USA; 2 Department of Radiology and Biomedical Imaging, University of California San Francisco, 500 Parnassus Ave, M-391, San Francisco, CA 94134 USA; 3 University of Buea, Buea, Cameroon; 4 Department of Biomedical Engineering, Marquette University, 1515 W Wisconsin Ave, Milwaukee, WI 53233 USA; 5 Department of BioHealth Informatics, School of Informatics and Computing, Indiana University - Purdue University Indianapolis, Office: 119 Walker Plaza (WK), 719 Indiana Avenue, Indianapolis, IN 46202 USA

**Keywords:** Open source, Radiology Information System, Enterprise imaging

## Abstract

Open-source development can provide a platform for innovation by seeking feedback from community members as well as providing tools and infrastructure to test new standards. Vendors of proprietary systems may delay adoption of new standards until there are sufficient incentives such as legal mandates or financial incentives to encourage/mandate adoption. Moreover, open-source systems in healthcare have been widely adopted in low- and middle-income countries and can be used to bridge gaps that exist in global health radiology. Since 2011, the authors, along with a community of open-source contributors, have worked on developing an open-source radiology information system (RIS) across two communities—OpenMRS and LibreHealth. The main purpose of the RIS is to implement core radiology workflows, on which others can build and test new radiology standards. This work has resulted in three major releases of the system, with current architectural changes driven by changing technology, development of new standards in health and imaging informatics, and changing user needs. At their core, both these communities are focused on building general-purpose EHR systems, but based on user contributions from the fringes, we have been able to create an innovative system that has been used by hospitals and clinics in four different countries. We provide an overview of the history of the LibreHealth RIS, the architecture of the system, overview of standards integration, describe challenges of developing an open-source product, and future directions. Our goal is to attract more participation and involvement to further develop the LibreHealth RIS into an Enterprise Imaging System that can be used in other clinical imaging including pathology and dermatology.

## Introduction

Radiology Information Systems (RIS) provide electronic support for the radiology workflow including scheduling, resource management, results storage and distribution, and billing. While proprietary RIS systems are common throughout developed countries, these solutions are frequently too expensive, or lack support in the developing world. Open-source solutions are therefore attractive in the developing world due to their low cost. While there are good open-source choices in several health IT spaces including electronic health record (EHR) and DICOM image archive, solutions for open-source RIS are nearly non-existent. Our search found two options: ThaIRIS [1], a project developed in 2016 without an active community. We also found a reference for HoruX [2] released in 2011 to work with Osirix [3], and no longer supported.

OpenMRS [4, 5] is an open-source electronic health record (EHR) system currently used in over 40 countries. The OpenMRS Atlas (atlas.openmrs.org) provides a self-reported overview of the different research, clinical, evaluation, and development implementations of OpenMRS. The EHR provides basic functionality including patient registration, demographic management, and a terminology dictionary using the Columbia International E-Health Laboratory (CIEL) [6] interface terminology that is mapped to ICD9, ICD10, LOINC, and SNOMED. The OpenMRS project started in 2004 as a multi-institution collaborative between the Regenstrief Institute, Partners in Health, and the Medical Research Council of South Africa primarily to build an open-source EHR platform that can be used for HIV/AIDS and TB care in low- and middle-income countries. Over the years, the project has evolved into a community of hundreds of contributors and organizations from across the world that have built EHR applications on the platform, which range from mobile-based data collection to telemedicine and health information exchanges. During this evolution, the project did not develop a clear governance model, but prioritized needs based on the organizations that provided the most number of contributors. It is only in the last couple of years, that a few EHR distributions based on OpenMRS have become their own projects and co-exist as separate brands of their own within and outside the OpenMRS community.

Since 2011, we have developed an open-source RIS system that was initially a small module for OpenMRS. After that, LibreHealth RIS was established as a standalone project under LibreHealth [7], which is an umbrella organization of multiple digital health systems including an electronic medical records system and a toolkit for development. The project has a project maintainer assisted by a co-maintainer, as well as a developer advocate that organizes contributions from multiple volunteer developers. The application is licensed under Mozilla Public License V2.0 (MPL) with a health disclaimer [8]. The health disclaimer is an addendum to MPL to disclaim liability related to privacy laws and healthcare regulations. An active community exists to support the RIS, actively collecting, documenting, and prioritizing user needs to guide development, and maximize use of limited resources.

This paper focuses on the evolution of open-source development of the LibreHealth RIS. We provide a historical and architectural overviews, including use of standards to ensure future scalability.

## LibreHealth RIS History and Architecture

### RIS Release Version 1 (2011–2013) [9]

The first version of the open-source RIS system was developed and released in 2011 and was available for public use until 2013. The development was led by two students from the University of Cauca, as part of a student project with supervision from one author (SP), an OpenMRS community mentor. The objective of developing the module was to improve the radiology workflow by managing orders, implementing roles in the department, scheduling studies, and providing a mechanism to view the images and generate a simple report. These tasks are described in detail below.

As mentioned above, RIS version 1 was created as a module for OpenMRS version 1.9.x. Within the OpenMRS data schema, each patient detail is recorded as an observation. Each observation is stored as a concept and answer pair, and with both ideally linked to the CIEL terminology dictionary. For example, a chest radiograph result is recorded with a concept with a unique ID/UUID and an answer type of text within the observation table.

**Fig. 1:**
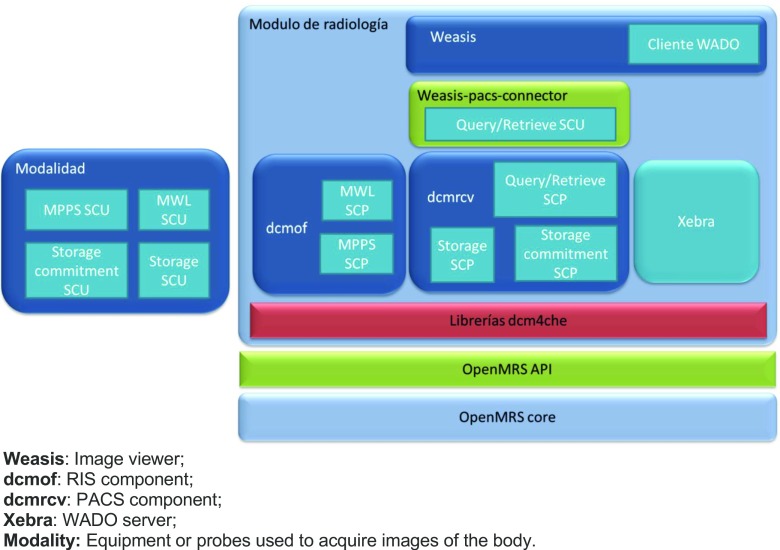
RIS v1 module architecture

Figure 1 shows an architecture overview of the RIS which used an image toolchain built with open-source components: Weasis image viewer [10], Xebra PACS database [11], and dcm4che DICOM [12] libraries. Version 1 provided three benefits: manage orders interface, study interpretation, and integrated image viewer.

#### Manage Orders

**Fig. 2:**
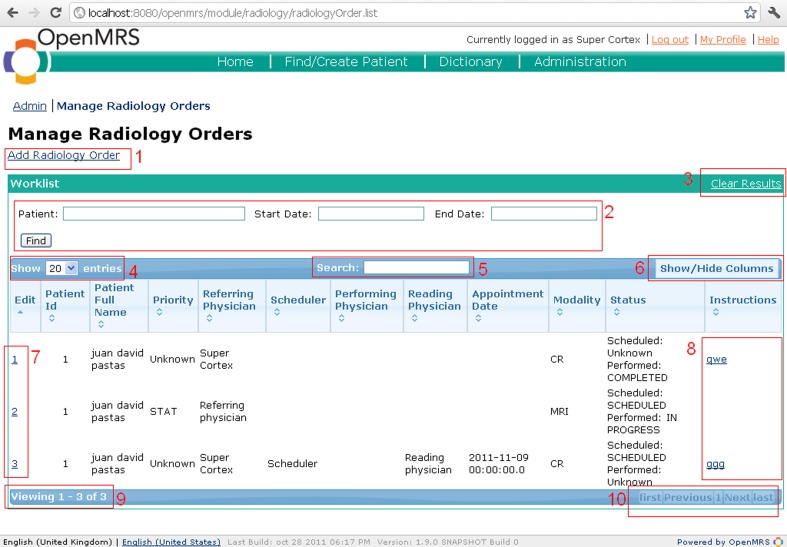
RIS release v1—Manage radiology orders

At this point of module development, there was no implementation of an order entry system within OpenMRS. A basic order entry system was developed to generate a modality worklist to communicate with PACS. For our testing and implementation, we used Xebra PACS [11], a lightweight medical imaging service. The module provided functionality to add, view, edit, or void radiology orders and also provided a user-customizable interface with flexible columns and filtering (Fig. 2).

The concept dictionary provides a list of tests that can be ordered. At the time of development, no RADLEX Playbook [13] or procedure codes (CPT) that were mapped to the OpenMRS concept dictionary were available for integration with the existing reference dictionary. We do not use CPT codes due to licensing terms from the American Medical Association that requires payment for code use. The manage orders interface was also specific to user role: for example, the referring physician was able to see scheduled and performed studies while the scheduler had additional fields to select an appointment slot for the patient.

#### Viewing Images

**Fig. 3:**
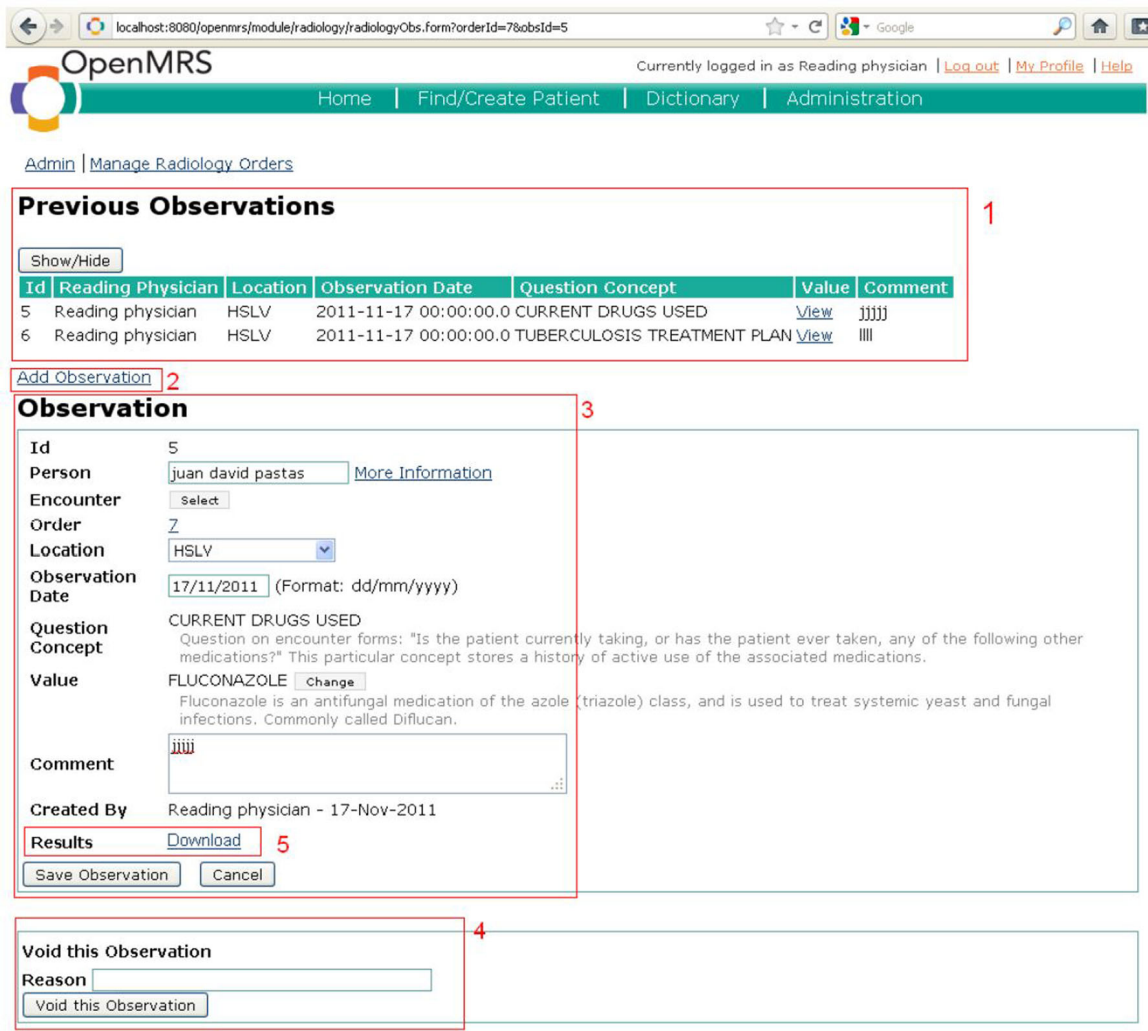
RIS release v1—Weasis image viewer controls

When a radiology study is marked complete, a download link is available, which when clicked, launched the Weasis image viewer. The Weasis viewer is broken into three panels: 1—image display, 2—show DICOM metadata, and 3—detailed information corresponding to the selected button (Fig. 3). A simple menu provided functionality to support measurements, manage display options and an image tool to support windowing, lookup tables, zooming, and rotation. One limitation of Weasis is that it requires Java to be installed and maintained on the computer used for image interpretation.

#### Study Interpretation

**Fig. 4:**
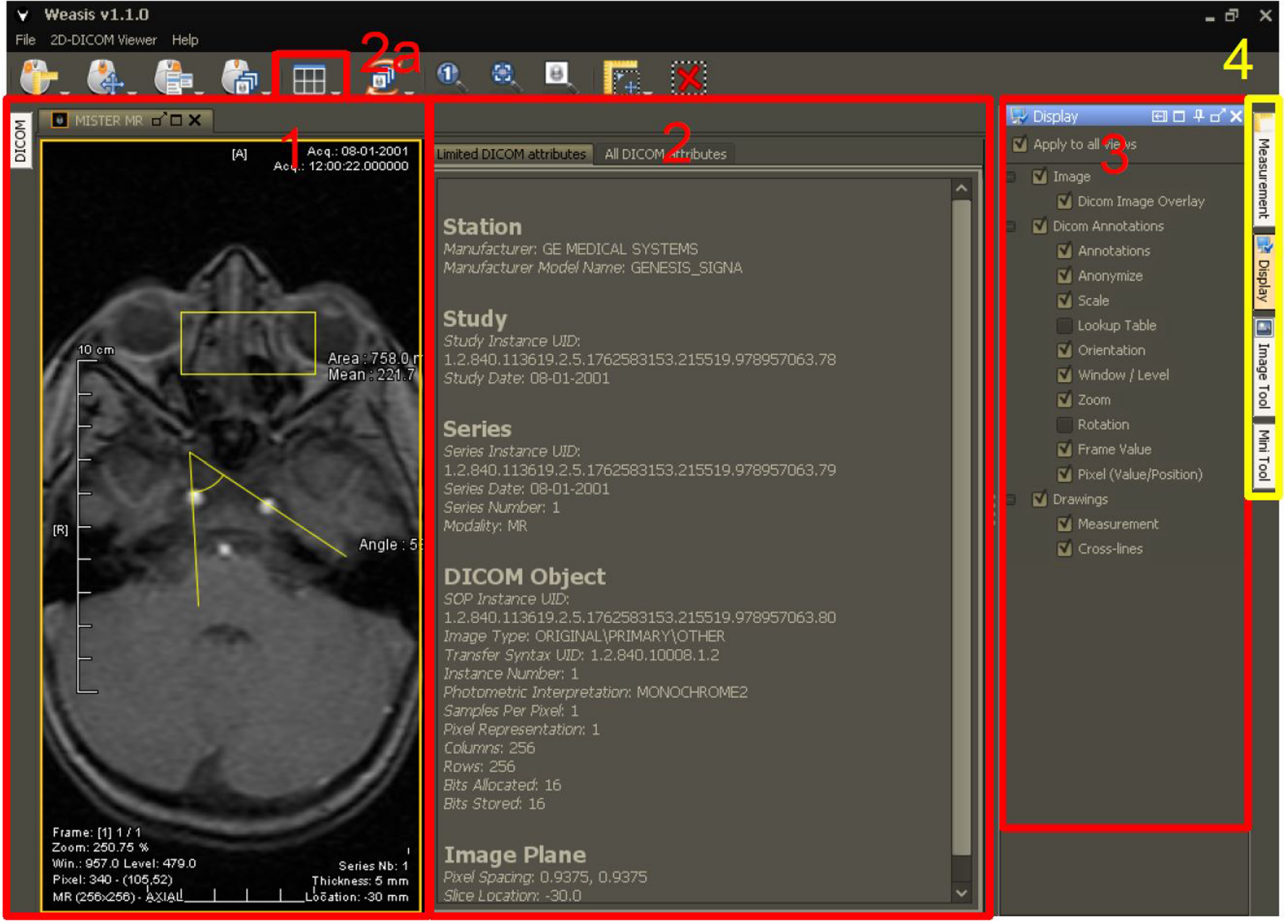
RIS release v1—Observation form used for radiology report creation

Once a study was marked completed within the RIS, the radiologist was able to create a new observation that captures the report interpretation as shown in Fig. 4. Reports were stored in the comment field as free-text. Templates and default reports were not available in v1. However, radiologists did have easy access to important clinical information under the Previous Observations heading.

Version 1 did not support trainee workflow, preliminary, or multimedia reports.

The code for this module is available here http://svn.openmrs.org/openmrs-modules/radiology/. No developer support is available for this version of the module, as we focus limited resources on subsequent versions.

### RIS Release Version 2 (2014–2016) [14]

**Fig. 5:**
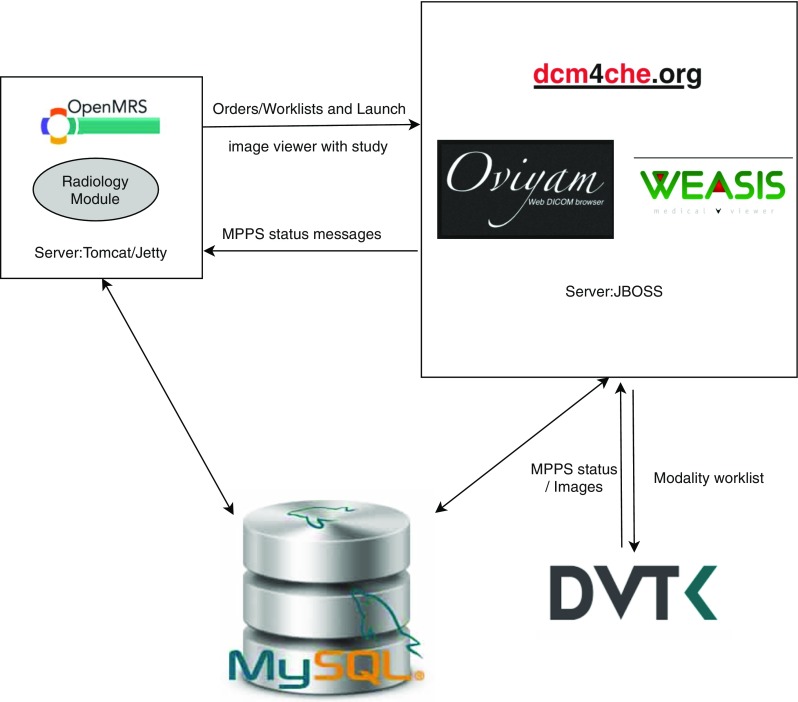
RIS version 2 architecture

Development of RIS v2 was focused on improving the backend functionality of the module including migrating the existing PACS connection away from Xebra to DCM4CHE [16], a widely used open-source PACS. RIS v2 would support image viewing with Oviyam in addition to Weasis. Oviyam [15], an open-source web-based image viewer, allowed us to move away from a client-side java dependency. As OpenMRS continued to meet our needs for basic functions, the second release was again developed as a module. Another reason to cut backwards compatibility to version 1 was to take advantage of improved OpenMRS module functionality, as well as decreasing developer overhead through new OpenMRS community development practices, primarily moving from ant to maven. Figure 5 shows a summary of the v2 RIS architecture.

Version 2 also improved manage orders functionality of version 1 including a new modality worklist that supported Hl7 [16] messaging using “dcmof” [17] and “hl7snd” [18] from the dcm4che toolkit.

#### Manage Orders

**Fig. 6:**
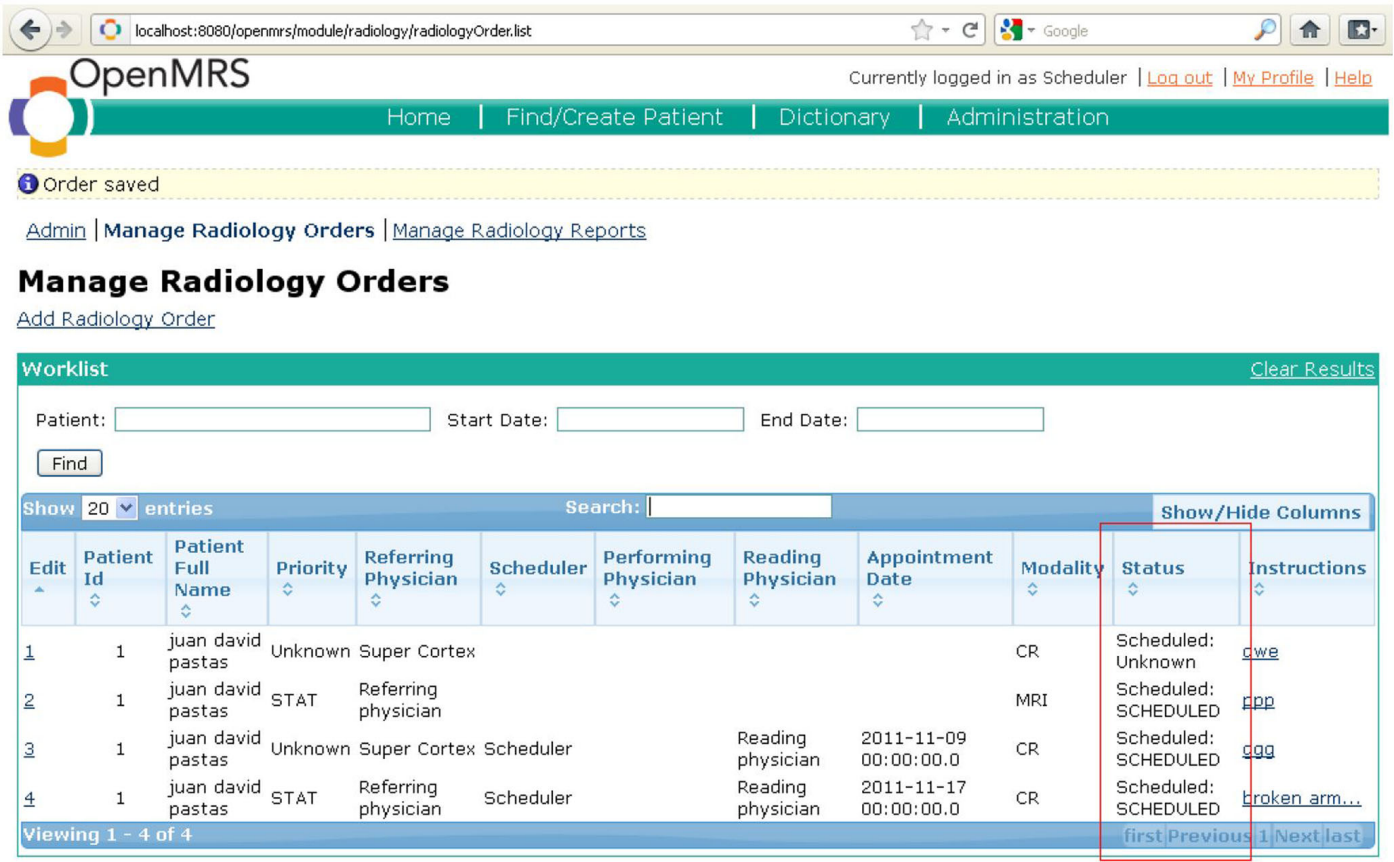
RIS version 2—Updated order page showing the modality worklist sync status as the last column on the view orders page

The bulk of the functionality from v1 was replicated in v2 using a new OpenMRS API for order entry. Additionally, orders entered into OpenMRS were sent to dcm4chee so that they could populate the modality worklist. In the radiology module, users were able to view the status of order synchronization (Fig. 6).

There were no changes to the reporting workflow for version 2. Radiologists still had free-text reports without defaults or templates. Clinical information was easily available on the reporting screen.

This module is available here https://github.com/openmrs/openmrs-module-radiology.

### RIS Release Version 3 (2017–Present) [19]

RIS v3 represents a change in the architecture of the previous releases and lacks backward compatibility with the older releases. The changes in RIS v3 were focused on improving the radiologist experience with improved worklists, reporting, and image display. We leveraged maturing standards including MRRT [20], adoption of structured reporting templates, migration of HL7 to FHIR [16], and DICOMWeb [21]. Weasis and Oviyam were replaced with Cornerstone, a new web-based open-source DICOM viewer. We also replaced the image archive functionality provided previously by dcm4chee with Orthanc [22] using DICOMWeb.

**Fig. 7:**
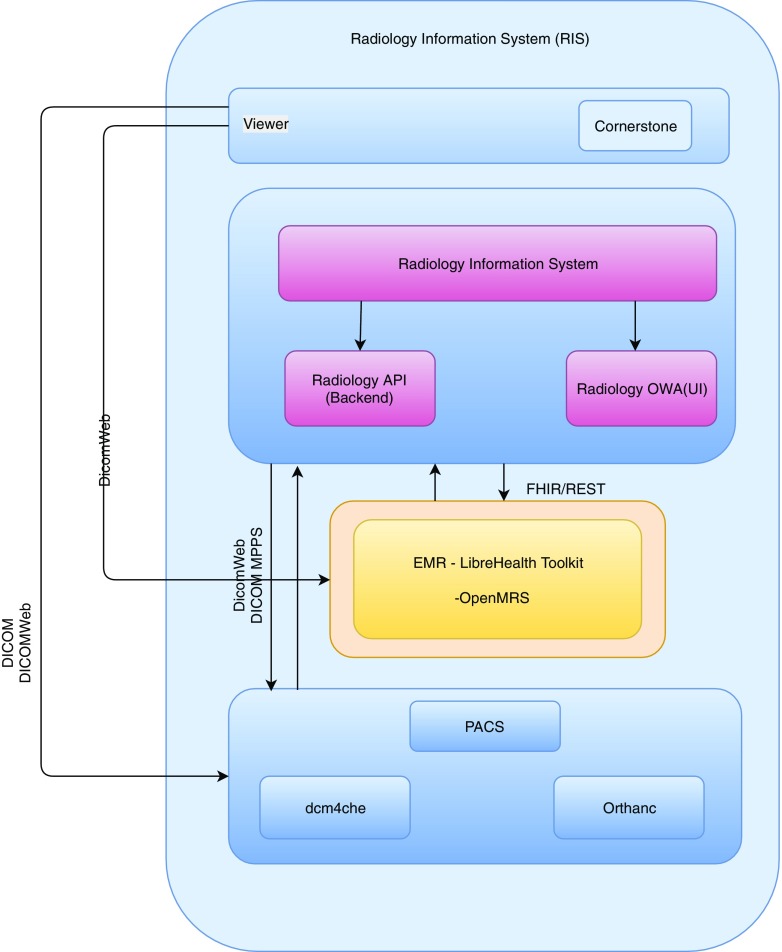
RIS version 3—Architecture showing integration of DICOM, DICOMWeb, and FHIR standards

Our decision to move to an HTML-based architecture was to overcome barriers with maintenance and support especially in limited resource settings where personnel may not be well trained to troubleshoot imaging systems in production. Moreover, an HTML-based workflow supports viewing across multiple devices including tablets and cell phones which have wide adoption in limited resource settings where we work (Fig. 7).

#### Managing Orders

In RIS v3, implementation of radiology orders is based on FHIR STU3 (v3.0.1) ProcedureRequest [16], which provides a record of a request to be planned, proposed, or performed. The ProcedureRequest, in turn, leads to either a Procedure or DiagnosticReport that can contain one or multiple Observations. The intent of our decision to adopt these four FHIR resources was to reduce future maintenance efforts and to not require backend database changes as data is stored in JSON documents. The core application needs to match with new FHIR API releases, but does not require backend database changes as data is stored in JSON documents. Moreover, there is an expanded scope of the ProcedureRequest standard beyond radiology to include endoscopies, surgeries, and other clinical interventions. This provides a platform to expand to enterprise imaging as the workflow for acquiring imaging from new use cases is already implemented in the base application.

#### Messaging and Modality Worklist

Implementation of HL7 messaging in RIS v2 was strictly tied to the radiology module requirements and was limited in utilization. Lack of a proper messaging queue complicated troubleshooting of errors when messages failed to reach the PACS system. In RIS v3, we implement DICOMweb to exchange REST order messages with Orthanc PACS and then render images in Cornerstone, an HTML-based image viewer.

#### Radiologist Workflow

**Fig. 8:**
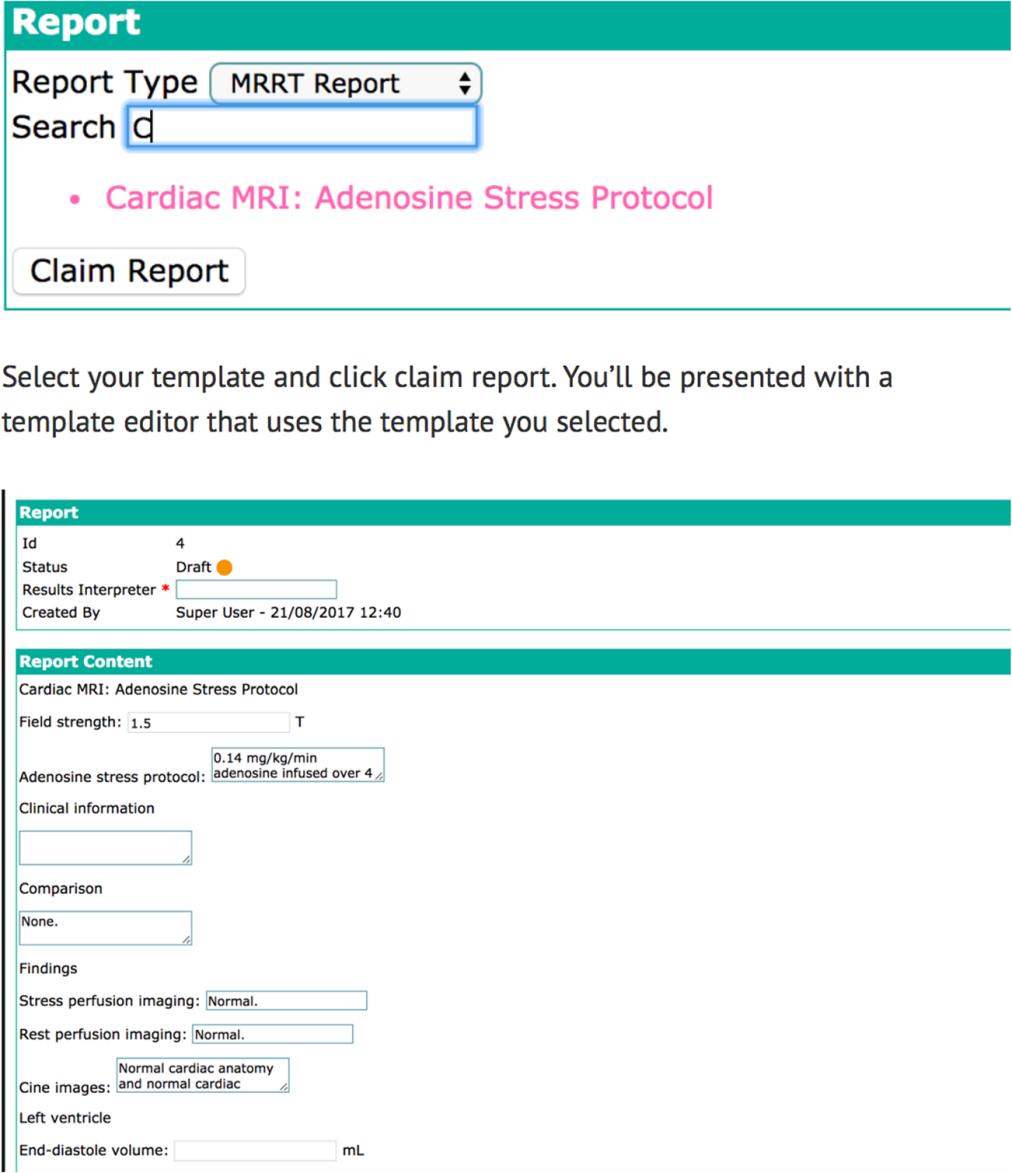
RIS version 3—Importing an IHE MRRT Cardiac MRI template for generating a radiology report

**Fig. 9:**
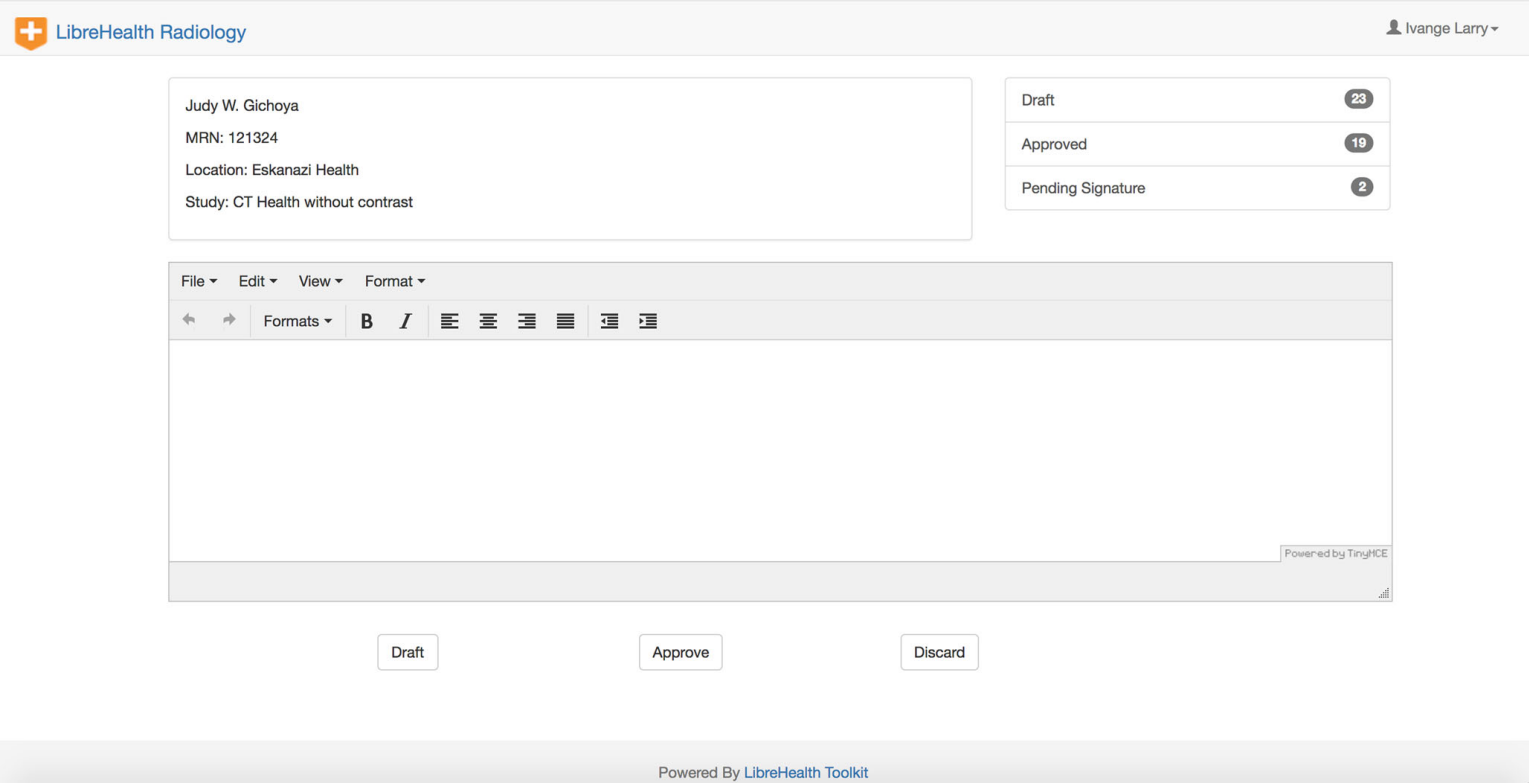
RIS version 3—New user interface workflow that provides for an editor to generate free-text report

RIS v3 presents a significant upgrade to the radiologist workflow supporting importing and use of IHE Management of Radiology Report Templates (MRRT) structured templates published on RSNA Radreport (www.radreport.org). Additionally, the radiologist trainee and staff workflow have incorporated queuing, approving, and preliminary reports for radiology studies. For example, in Fig. 8, the user selects a cardiac MRI protocol from the available RadReport MRRT templates. Subsequently, the template is rendered as an HTML page that can be filled by the user and saved to generate a radiology report for that study. If an appropriate MRRT template is not available, RIS v3 provides the user with a free-text report template, as shown in Fig. 9.

An additional feature developed shows a reading list accessible by multiple radiologists that supports study priorities.

In Fig. 8, the imported templates renders as an HTML template for with fields for structured report generation.

#### User Interface Changes

Based on the decision to separate the backend API and the frontend user interface, we have a new JavaScript interface that is dependent on web components. The FHIR server design change allows developers to create new user interfaces when desired while retaining the existing user interfaces when end users do not wish to make a complete system migration.

The development of this module is ongoing with project code available at https://gitlab.com/librehealth/lh-radiology with a planned beta release in early 2018.

## Discussion

We describe our experience developing an open-source RIS system across multiple systems and how we have adopted standards to guide future development. In developing open-source systems, we have encountered challenges from incorporating packages that became unsupported, as in the case of Xebra PACS. Moving forward, we study the health of an open-source community before integrating new components, using tools like Community Health Analytics Open Source Software Project [25] (CHAOSS) that provide objective and repeatable metrics for community contributions and development. CHAOSS provides tools such as Grimoirelab, Prospector, and Cregit that helps gather detailed data from code, mailing lists, and stackoverflow about the activity in an open-source community.

Moreover, adoption of standards like FHIR minimizes the future change effort to keep up with core EMR platform changes and now requires updating the FHIR toolkit libraries when new standards are released. Decoupling the backend and frontend allows cross-platform implementation as long as standards are adhered to, as well as aids in improving migration across different systems. Adopting an architecture that is based on DicomWeb provides a strategic advantage in terms of software development, implementation, and support. Each application page developed is driven by a user story, for example “A technologist will need to complete the studies performed.” This user story is then designed and the final wireframe decoupled into individual web components that are developed for the frontend. Through such iterative software development, we are developing a library of most commonly used web components that are easily adopted and used for new applications. DicomWeb allows us to have a complete html-based system from the EMR, reporting tools, and image viewing which lowers the burden of adoption and use in developing countries where there are few imaging informatics specialists.

## Conclusion

In this paper, we discuss the development of an open-source radiology system, and how an open-source community can serve as ground for innovation to accelerate adoption of new standards as well as modernize the imaging informatics systems architecture. We welcome readers to join in our community to share opinions and feedback to accelerate our development and growth.
